# The 4T and 7T introgressions from *Amblyopyrum muticum* and the 5A^u^ introgression from *Triticum urartu* increases grain zinc and iron concentrations in Malawian wheat backgrounds

**DOI:** 10.3389/fpls.2024.1346046

**Published:** 2024-07-16

**Authors:** Veronica F. Guwela, Moses F. Maliro, Martin R. Broadley, Malcolm J. Hawkesford, James M. Bokosi, Surbhi Grewal, Benedict Coombes, Anthony Hall, Caiyun Yang, Mike Banda, Lolita Wilson, Julie King

**Affiliations:** ^1^ School of Biosciences, University of Nottingham, Loughborough, United Kingdom; ^2^ Rothamsted Research, Harpenden, United Kingdom; ^3^ Lilongwe University of Agriculture and Natural Resources, Lilongwe, Malawi; ^4^ Earlham Institute, Norwich Research Park, Norwich, United Kingdom

**Keywords:** micronutrients, biofortification, zinc, iron, introgression, phenotyping, genotyping

## Abstract

Micronutrient deficiencies (MNDs) particularly zinc (Zn) and iron (Fe) remain widespread in sub-Saharan Africa (SSA) due to low dietary intake. Wheat is an important source of energy globally, although cultivated wheat is inherently low in grain micronutrient concentrations. Malawian wheat/*Am. muticum* and Malawian wheat/*T. urartu* BC_1_F_3_ introgression lines, developed by crossing three Malawian wheat varieties (*Kenya nyati*, *Nduna* and *Kadzibonga*) with DH-348 (wheat/*Am. muticum*) and DH-254 (wheat/*T. urartu*), were phenotyped for grain Zn and Fe, and associated agronomic traits in Zn-deficient soils, in Malawi. 98% (47) of the BC_1_F_3_ introgression lines showed higher Zn above the checks Paragon, Chinese Spring, *Kadzibonga*, *Kenya Nyati* and *Nduna*. 23% (11) of the introgression lines showed a combination of high yields and an increase in grain Zn by 16-30 mg kg ^-1^ above *Nduna* and *Kadzibonga*, and 11-25 mg kg ^-1^ above *Kenya nyati*, Paragon and Chinese Spring. Among the 23%, 64% (7) also showed 8-12 mg kg ^-1^ improvement in grain Fe compared to *Nduna* and *Kenya nyati*. Grain Zn concentrations showed a significant positive correlation with grain Fe, whilst grain Zn and Fe negatively and significantly correlated with TKW and grain yield. This work will contribute to the efforts of increasing mineral nutrient density in wheat, specifically targeting countries in the SSA.

## 1 Introduction

Micronutrient deficiencies (MNDs) remain a global challenge, affecting an approximated 2 billion people worldwide (White and Broadley, 2009; World Health, 2009). Zinc (Zn) and iron (Fe) deficiencies are widespread in low-income countries, particularly in sub-Saharan Africa (SSA) and South-east Asia (Gupta et al., 2020). Inadequate intake and bioavailability of these elements in diets remain the major reasons for increased deficiency risks (Caulfield and Black, 2002; Maret and Sandstead, 2006; Prasad et al., 2014). A high dependence on cereal diets and inability to afford foods that are rich in essential micronutrients for a majority of people in SSA has resulted in risk deficiencies of up to 96%, with a number of countries falling above 50% (Kumssa et al., 2015). In Malawi for instance, Zn deficiency risk is at ~60% of the population with most households having deficiency risks in the range of 50-75% (Joy et al., 2014; NSO, 2016; Likoswe et al., 2020). It is estimated that malnutrition results in an annual economic burden of 10.3% of Malawi’s gross domestic product (UNICEF, 2019). Food based approaches, particularly food fortification, agronomic biofortification and genetic biofortification of staple crops, were identified as strategies to combat Zn deficiencies globally (Gibson and Ferguson, 1998; Graham et al., 1999; Welch, 2002; Bouis, 2003; Velu et al., 2014). However, food fortification and agronomic biofortification programs are more feasible in developed countries compared to the least-developed, low-income countries, owing to low accessibility and cost of industrially processed food and micronutrient rich fertilisers (Horton, 2006; Cakmak, 2008; Gomez-Galera et al., 2010). In contrast, genetic biofortification, which aims at enhancing grain micronutrient concentration and substances that promote nutrient bioavailability through plant breeding (Velu et al., 2014; Bouis and Saltzman, 2017), is a viable and cost-effective approach for delivering essential micronutrients to low-income countries.

Wheat is an important staple crop providing more than 20-25% daily calorie intake in Africa (FAO, 2019). In recent years, demand for wheat and wheat products in SSA has substantially increased and is projected to increase further in the immediate future (Shiferaw et al., 2011; Mason et al., 2015; Guwela et al., 2021). Previous work has shown that genetic variation for grain Zn and Fe concentration in most of the cultivated wheat is not enough to meet the estimated average requirement (EAR) for both children and adults of reproductive and non-reproductive age. In contrast, wheat progenitor species and other wild relatives in the wheat secondary and tertiary gene pools have revealed a substantial genetic variation for grain concentrations of Zn, Fe and other essential minerals (Chhuneja et al., 2006; Rawat et al., 2009; Neelam et al., 2011; Rawat et al., 2011; Tiwari et al., 2015). Through introgression breeding, genetic variation from wheat progenitors and wild relatives can be successfully transferred to modern cultivated wheat (Anderson, 1949; Thórsson et al., 2001) to tap some useful genetic variability for wheat improvement. Several studies have shown higher grain Zn and Fe concentrations in introgression lines developed from cultivated hexaploid/tetraploid wheat and several wild species compared to their modern cultivated wheat parents (Rawat et al., 2009; Tiwari et al., 2010; Neelam et al., 2011; Wang et al., 2011; Farkas et al., 2014). For example, wheat/wild relative derivatives from *Ae. kotschyii* and *Ae. peregrina* have shown up to a five-fold increase in grain Zn concentration above their recurrent parents (Tiwari et al., 2010; Neelam et al., 2011; Rawat et al., 2011). Progenitor species, particularly *Ae. tauschii* have also shown to increase grain Zn and Fe concentrations by 20-40% compared to local varieties (Singh et al., 2017).

Thus, the transfer of genetic variation from wheat wild relatives to cultivated wheat through introgression of chromosome segments from wheat wild relatives offers a useful approach for improving the nutritional quality of wheat to the target levels required for improving human nutrition. Pre-breeding efforts have resulted in the successful transfer of a number of progenitor and wild relative chromosomes from the genus, *Triticum*, *Aegilops*, *Amblyopyrum* and *Thinopyrum* (King et al., 2018; Grewal et al., 2018b; King et al., 2019; Grewal et al., 2021). Mineral analysis of some of the pre-breeding materials have shown substantial variation in grain Zn and Fe (Guwela, 2023) and these may be useful sources for transferring the introgressions into other adapted wheat backgrounds.

The breeding targets for grain Zn and Fe concentration in wheat were set at an additional 12 and 22 mg/kg from 25 and 30 mg/kg respectively (Bouis et al., 2011; Bouis and Saltzman, 2017). These targets were set to meet 60-80% of EAR for preschool children (4–6 years old) and for non-pregnant and non-lactating women of reproductive age (Bouis and Saltzman, 2017). Previously, rye translocations in a Pavon 76 wheat background significantly increased grain zinc concentrations above the recurrent parent (Velu et al., 2019). In CIMMYT, the use of *Triticum aestivum* ssp. spelta- and *Triticum turgidum* ssp. dicoccum-based synthetics have resulted in the release of varieties with 20-40% higher Zn levels compared to local varieties (Singh et al., 2017; Guzman et al., 2019; Velu et al., 2019). Similarly, HarvestPlus Yield Trials (HPYT) of CIMMYT biofortified wheat varieties released in Nepal showed a combination of high yields and high grain Zn and Fe concentrations above the local checks (Thapa et al., 2022).

This paper describes the transfer of *Am. muticum* (TT) and *T. urartu (*A^u^A^u^ *)* introgressions, from doubled haploid introgression lines into Malawian wheat varieties to achieve increased grain mineral concentration.

## 2 Materials and methods

### 2.1 Germplasm

The introgression lines used in this study were developed by crossing DH-348 and DH-254 with three Malawian wheat varieties (*Kadzibonga*, *Nduna* and *Kenya nyati*). The Nottingham Wheat Research Centre (WRC), at the University of Nottingham previously developed the DH lines. Briefly, DH-348 was developed by pollinating hexaploid wheat cv. Pavon 76 with *Am. muticum* accession 2130012 and *T. urartu* DH-254 was developed from a cross between hexaploid wheat cv. Chinese Spring (*ph1/ph1*) and *T. urartu* accession 1010002 (King et al., 2017; Grewal et al., 2018a). The F_1_ interspecific hybrid carrying the *Am. muticum*/wheat and the *T. urartu*/wheat recombinant chromosome were backcrossed as females to Paragon up to BC_3_, which was used to produce the DH lines (King et al., 2019; Grewal et al., 2021). The three Malawian wheats, *Kenya Nyati*, *Kadzibonga* and *Nduna* were obtained from stocks retained at Lilongwe University of Agriculture and Natural Resources (LUANAR) Malawi. The three Malawian varieties represent the most widely cultivated wheat varieties in Malawi.

### 2.2 DNA sequencing

For the crossing program, two parental DH lines (DH-254 and DH-348) were sequenced to determine their genetic makeup, with a target on the size and site of introgression of the alien chromosome segments. Genomic DNA (deoxyribonucleic acid) was collected from 2-week-old leaf samples. Extraction was performed using extraction buffer (0.1 m Tris–HCl (pH 7.5), 0.05 m EDTA (pH 8.0), 1.25% SDS). Library preparation and DNA sequencing was performed by the Novogene (UK) Company Limited. The DNA sample used for library preparation was prepared following the manufacture’s recommendations of NEBNext^®^ DNA Library Prep Kit (New England BioLabs, US). Index codes were added to each sample. Briefly, the genomic DNA was randomly fragmented to size of 350 bp. DNA fragments were end polished, A-tailed, ligated with adapters, size selected, and further PCR enriched. Then polymerase chain reaction (PCR) products were purified (AMPure XP system), followed by size distribution by Agilent 2100 Bioanalysis (Agilent Technologies, CA, USA), and quantification using real-time PCR. The library was then sequenced for 10x whole genome sequencing (WGS) on NovaSeq 6000 S4 flow cell with PE150 strategy.

### 2.3 Generating a segregating population

Hybridisation of the donor (DH-348 and DH-254) and the reciprocal parents (*Nduna*, *Kadzibonga* and *Kenya nyati*) was performed in both directions (as both male and female). In total, six cross combinations were made for each of the DH lines. Selected F_1_ interspecific hybrids were backcrossed to respective recurrent parents to obtain the BC_1_ population, which was selfed up to BC_1_F_3_. Germination was followed by 4 weeks vernalisation (6°C and photoperiod for 12 hours) seven day after sowing. The plants were left under glasshouse conditions with the photoperiod set at 25°C, light at 16 hours and 8 hours dark. Emasculation was done before the spikes completely emerged from the flag leaf. Emasculated spikes were covered with glassine bags following removal of the anthers. Pollination was done two days after emasculation.

### 2.4 Genotyping

To characterise the introgression lines, genomic DNA of the BC_1_ and BC_1_F_1_ populations was extracted in a 96 well plate from leaf samples collected from 10 day old seedlings (Thomson and Henry, 1995). Extraction was performed using template preparation solution (TPS) buffer and isopropanol. Malawian wheats, *Kadzibonga*, *Kenya nyati* and *Nduna* alongside wheat/*Am. muticum*, DH 348, wheat/*T. urartu*, DH-254 *Am. muticum* accession 2130012 and *T. urartu* accession 1010002 were used as controls. The KASP assays comprised of two allele specific primers and one common reverse primer. A final reaction volume of 5 μl, which included 1ng genomic DNA, 2.5 μl KASP reaction mix (ROX), 0.068 μl primer mix and 2.43 μl nuclease free water Primer mix, was dispensed into the 386 well plates using Gilson pipette max 268 (Gilson, INC. 3000 Parmenter St. Middleton, WI 53562). Plates were sealed with optical quantitative polymerase chain reaction (qPCR) seals (Sarstedtstr AG & Co. KG, Numbrecht, Germany) following a brief centrifuge. Genotyping was done using ProFlex PCR system (Applied Biosystems by Thermo Fisher Scientific). PCR conditions were set as 15 min at 94°C; 10 touchdown cycles of 10 s at 94°C, 1 min at 65–57°C (dropping 0.8°C per cycle); and 35 cycles of 10 s at 94°C, 1 min at 57°C.

### 2.5 Genomic *in-situ* hybridisation

To validate the presence of the chromosome segments in the introgression lines, genomic *in-situ* hybridisation (GISH) was performed following a protocol described by Kato et al. (2004) and King et al. (2017). Genomic DNA was extracted from *Am. muticum* and the three progenitors of bread wheat: *T. urartu*, *Ae. speltoides*, and *Ae. tauschii* using an extraction buffer (0.1 M Tris-HCl, 0.05 m EDTA and 1.25% SDS). Genomic DNAs of *Am*. *muticum, T. urartu*, *Ae*. *tauschii* and *Ae*. *speltoides* were labelled by nick translation with ChromaTide Alexa Fluor 546-14-dUTP (Alexa Fluor-546), ChromaTide Alexa Fluor 488-5-dUTP (Alexa fluor-488) [Thermo Fisher Scientific (Invitrogen), Waltham, MA, United States] and Alexa Fluor 594-5-dUTP (Alexa fluor-594) [Thermo Fisher Scientific (Invitrogen), Waltham, MA, United States] and ChromaTide Alexa 405 dUTP, respectively. Metaphase spreads were prepared from root tips using a nitrous oxide-enzymatic maceration method. Malawian wheat/*Am. muticum* slides were probed using a probe mixture containing 1.5μl of *T. urartu*, 1.5μl *Ae. speltoides*, 2μl *Ae. tauschii* and 0.3μl *Am. muticum* labelled genomic DNA in 2 × SSC and 1 × TE buffer (pH 7.0) to a final volume of 10μl per slide. Malawian wheat/*T. urartu* slides were probed using a similar probe mixture with the exception of *Am. muticum* genomic DNA. Slides were counterstained with Vectashield mounting medium with 4-6-diamidino-2phenylindole dihydrochloride (DAPI). Analysis was done using a Zeiss Axio ImagerZ2 upright epifluorescence microscope (Carl Zeiss Ltd, Oberkochen, Germany) with filters for DAPI (Ex/Em 358/461 nm, blue), Alexa Fluor 488 (Ex/Em 490/520 nm, green), Alexa Fluor 594 (Ex/Em 590/615 nm, red) and Alexa Fluor 546 (Ex/Em 555/570 nm, yellow). Photographs were taken using a MetaSystems Coolcube 1 m CCD camera.

### 2.6 Field phenotyping

#### 2.6.1 Soil sampling, preparation and analysis

A composite soil sample was collected on each block at the trial site before trial establishment. Soil samples were air-dried, crushed with a pestle and mortar before passing them through a 2 mm sieve. 200 grams of each of the composite soil sample was transferred into zip-loc bags, labelled and shipped to the University of Nottingham for analysis. Soil pH was determined following suspension of 5 g of soil sample into 12.5 ml Milli-Q water (18.2 MΩ cm; 1:2.5 m/v). Total nitrogen (N) was measured using the Kjeldahl method (Kjeldahl, 1883). Organic matter was determined using the Walkley and Black method (Walkley and Black, 1934). Extractable soil Zn and Fe were determined by the diethylene triamine penta-acetic acid (DTPA) extraction method (Lindsay and Norvell, 1978) followed by multi-element analysis with ICP-MS. Available phosphorus (P) and K were measured using the Mehlich- 3 extraction (Mehlich, 1984).

#### 2.6.2 Experimental design, trial management and field data collection

The experiment was conducted in the winter of 2022 (May to October). Wheat lines were grown under field conditions in an optimally irrigated environment at LUANAR (14.18’S 33.76’ E) in Lilongwe, Malawi. Forty-eight BC_1_F_3_ introgression lines (11 Malawian wheat/*T. urartu* DH-254 and 37 Malawian wheat/*Am. muticum* DH-348) were planted alongside three Malawian wheat varieties (*Kadzibonga*, *Kenya nyati* and *Nduna*), two DH lines (DH-348 and DH-254) and Paragon, Pavon 76 and Chinese spring, in a randomised complete block design (RCBD) with three replicates. Plots were 2 m^2^ each, with six rows spaced at 0.15 m. Plot spacing was 0.30 m and block spacing was 1.0 m. Basal dressing fertiliser 23N:10P:5K +6S +1Zn (SuperFert Fertilisers, Harare, Zimbabwe) was applied 14 days after planting at a rate of 200 kg N/ha. Three weeks later, Urea (46% N) was applied as top dressing at a rate of 100 kg N/ha. Basal and top dressing were applied according to the Malawi guide to agriculture production (MoAI, 2020) guidelines. The Malawi government recently approved the NPK (23:10:5 + 6S +1Zn) basal fertiliser with 1% Zn due to severe deficiencies (< 2 mg kg^-1^) of plant available soil Zn across the country (IFDC et al., 2018). Thus, all basal fertiliser blends for selected cereals and legumes in Malawi have 1% Zn. First weeding was done 4 weeks after planting and subsequent weeding as soon as weeds appeared. Insect pests were controlled by applying Profex Super (Profencfos 40% + Cypermenthrin 4% EC –Kewalram Chanrai group). Data collected included days to heading (DH), days to flowering (DF), days to maturity (DM), plant height, thousand kernel weight (TKW) and grain yield. Grain yield was converted from g/m^2^ to kg/ha. Plant height and number of tillers were collected from five randomly selected plants in the net plot, to get an average of both.

### 2.7 Grain sample digestion and multi-elemental analysis

Grain samples were digested using a hot block acid digestion system (Anton Paar Gmbh, Graz, Austria) as described by Gashu et al., 2021. Approximately 0.4 g of each of the grain samples along with certified reference material (wheat flour 1567b-CRM) and laboratory reference material (Paragon wheat-LRM) were digested using a Multicube 48 digestion block (Anton Paar Gmbh, Graz, Austria). Two operational blanks were added in each run. The digestion block was set at 105°C for 2h. Samples were diluted with milliQ water (18.2 MΩ cm; Fisher Scientific UK Ltd, Loughborough, UK) up to 50 mL. Grain multi-element analysis was undertaken using inductively coupled plasma mass spectrometry (Thermo Fisher Scientific iCAPQ, Thermo Fisher Scientific, Bremen, Germany) as described by Gashu et al. (2021) and Khokhar et al., 2018. A total of 189 grain samples including blanks, CRMs and LRMs were analysed. The Zn and Fe specific recovery from CRMs from grain samples was 99 and 97% respectively. The limit of detection (LOD) values for grain Zn and Fe were 0.7 and 2.2 respectively.

### 2.8 Data analysis

The Earlham Institute bioinformatics pipeline (Coombes et al., 2022) was used to analyse the sequencing data. Florescence detection and data analysis of KASP reactions was performed using Quant Studio Design and Analysis Software V1.5.0 (Applied Biosystems by Thermo Fisher Scientific). GISH analysis was carried out using Meta Systems ISIS and Metafer software (Metasystems GmbH, Altlussheim, Germany). For field data, one-way analysis of variance (ANOVA) was performed using GenStat for windows statistical package, version 21 (VSN, 2022). A *post-hoc* test was performed using Tukey’s HDS test. Pearson correlation analyses were performed in RStudio (version 4.1.3), and correlation heatmaps were created using the same software (RStudio Team, 2022). The statistical linear model considered the response Y_ij_ of the jth treatment in the ith replication expressed as:


$$Yij=µ+\beta i+\tau i+e_{ij}$$

where *μ* is the grand mean of all genotypes, *β*_i_ is the block effect, τ*_j_* is the effect of the jth treatment (genotype) and *e_ij_* is the average experimental error.

## 3 Results

### 3.1 Sequencing and GISH analysis of the parental lines

**Figure 1:**
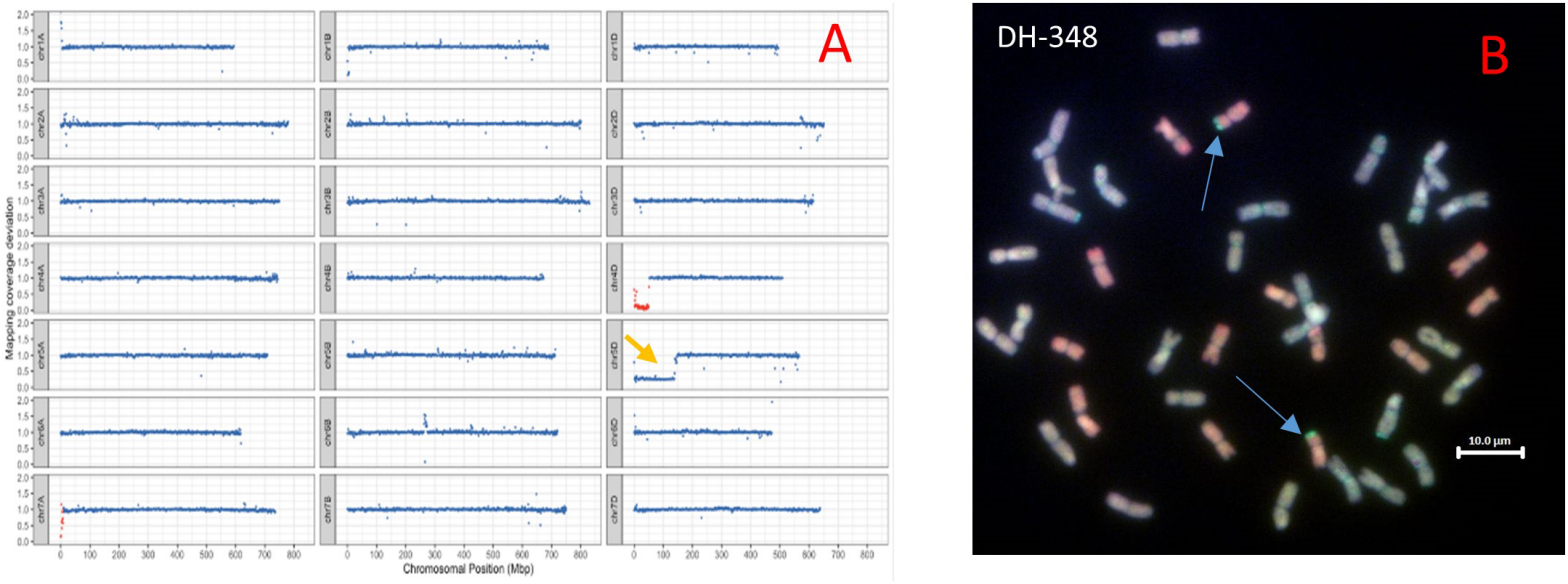
**(A)** Sequencing visualisation of DH-348 showing the *Am. muticum* segment introgressed on wheat Chr 4D and on Chr 7A (*Am. muticum* chromosomes in red blocks, wheat chromosomes in blue blocks), and a monosomic deletion on wheat Chr 5D (yellow arrow). **(B)** GISH image of metaphase spreads from roots of DH-348 showing the A, B, D and T genomes (A genome - green, B genome - blue, D genome – red, T genome - gold). The blue arrows indicate the site of *Am. muticum* introgressions.

The sequence reads from the parental lines, hexaploid wheat cvs. Chinese Spring and Paragon, and *Am. muticum* DH-348 were mapped to the wheat reference genome assembly cv. Chinese Spring RefSeq v.1.0 (IWGSC et al., 2018). Whole genome sequence analysis of *Am. muticum* DH-348 revealed the presence of two *Am. muticum* segments on wheat chromosomes (Chr) 4D and 7A as shown in the drop in read coverage (red blocks) in **Figure 1A**. Analysis of the size of the introgressed segments showed that the segment on Chr 4D is bigger (51.2 Mbp) compared to the segment on Chr 7A (9.1 Mbp). Sequence analysis also revealed a monosomic deletion on the short arm of Chr 5D. GISH analysis of *Am muticum* DH-348 (**Figure 1B**) partially validated the sequencing results, as it showed a pair of recombinant chromosomes with a large D chromosome (red) and a small T segment (gold) at the distal end of the D chromosome. The *Am. muticum* segment visible from the GISH metaphase spread is likely from Chr 4D as the segment on 7A is too small to be detected by GISH.

**Figure 2:**
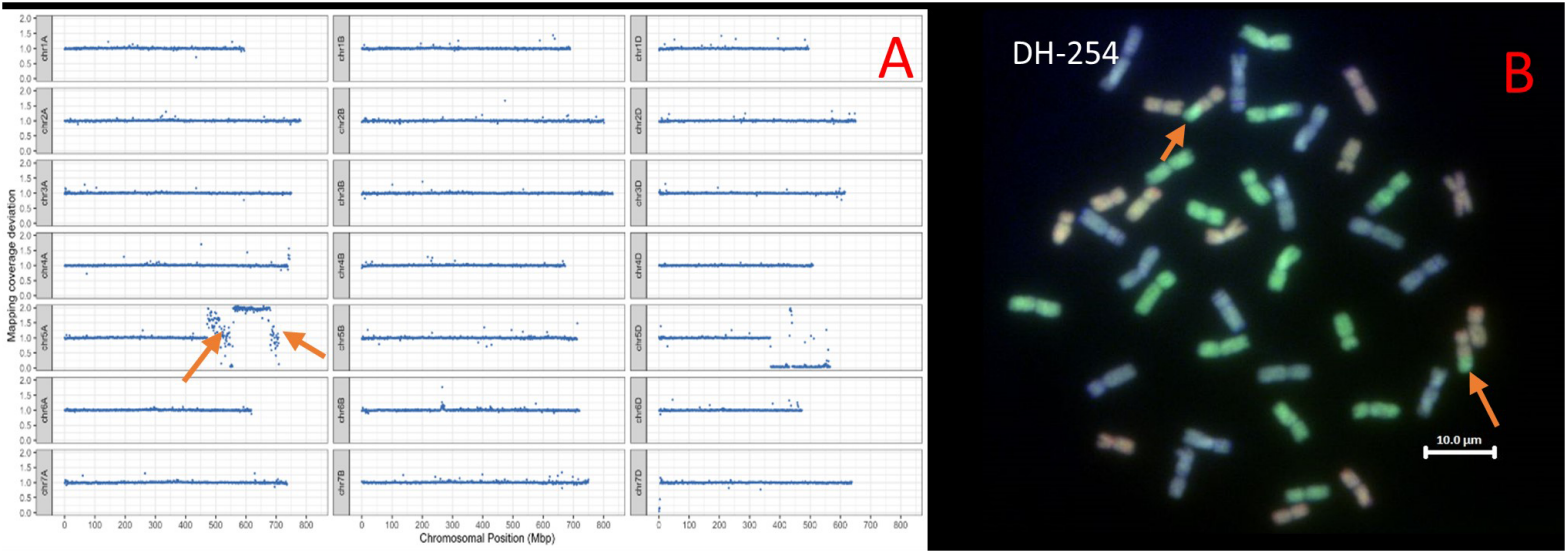
**(A)** Sequencing visualisation of DH-254 showing *T. urartu* segments introgressed on wheat Chr5A (shown by orange arrows) and the 5D-5A intergenomic recombination shown by the drop in chromosome block on chromosome 5D. **(B)** GISH image of metaphase spreads from roots of DH-254 showing the A, B and D genomes (A genome - green, B genome - blue, D genome – red). Orange arrows show the 5A-5D recombinant chromosomes.

Analysis of *T. urartu* DH-254 showed two segments of *T. urartu* recombined with the 5A chromosome of wheat. The segments were 76.40 and 28.77 Mbps. Sequence analysis also showed that a portion of chromosome 5A had duplicated and translocated to Chr 5D to replace the 5DL chromosome portion (**Figure 2A**). GISH analysis did not validate the presence of the 5A^u^ segments translocated to the 5A chromosome of wheat (**Figure 2B**) because the probe used for detecting the A genome of wheat is prepared from the wheat progenitor, *T. urartu* and thus in wheat/*T. urartu* introgression lines, the probe detects both the A and A^u^ genomes (Grewal et al., 2021).

### 3.2 Malawian wheat/*T*. *urartu* DH-254 and Malawian wheat/*Am*. *muticum* DH-348 segregating populations

In the initial round of crossing, 262 and 120 F_1_ seeds were obtained for the Malawian wheats/*Am. muticum* DH-348 and Malawian wheats/*T. urartu* DH-254 combinations, respectively. Backcrossing selected F1 seeds generated 362 and 190 BC_1_ seeds, and Self-fertilisation of the BC_1_ plants generated 11,058 Malawian wheat/*Am. muticum* DH-348 and 5,300 Malawian wheat/*T. urartu* DH-254 BC_1_F_2_ seeds. Further self-fertilisation/bulking of the seeds resulted in the generation of 46,950 Malawian wheat/*Am. muticum* DH-348 and 16,535 Malawian wheat/*T. urartu* DH-254 BC_1_F_3_ seeds. Among the Malawian wheat/*T. urartu* DH-254 cross combinations, three combinations (*T. urartu* DH 254 ×Kadzibonga, Kadzibonga ×*T. urartu* DH 254 and Nduna × *T. urartu* DH 245) were lost at F_1_ and BC_1_F_2_ due to the inability to produce seed, and failure of the selected seeds to germinate, respectively. Among the Malawian wheat/*Am. muticum* DH-348 cross combinations, only one combination (*Am. muticum* DH 348 × Kenya nyati) was lost at BC_1_F_2_ due to failure of the selected seeds to germinate.

### 3.3 Molecular and cytogenetic characterisation of Malawian wheat/*Am. muticum* BC_1_ and BC_1_F_1_ plants

**Figure 3:**
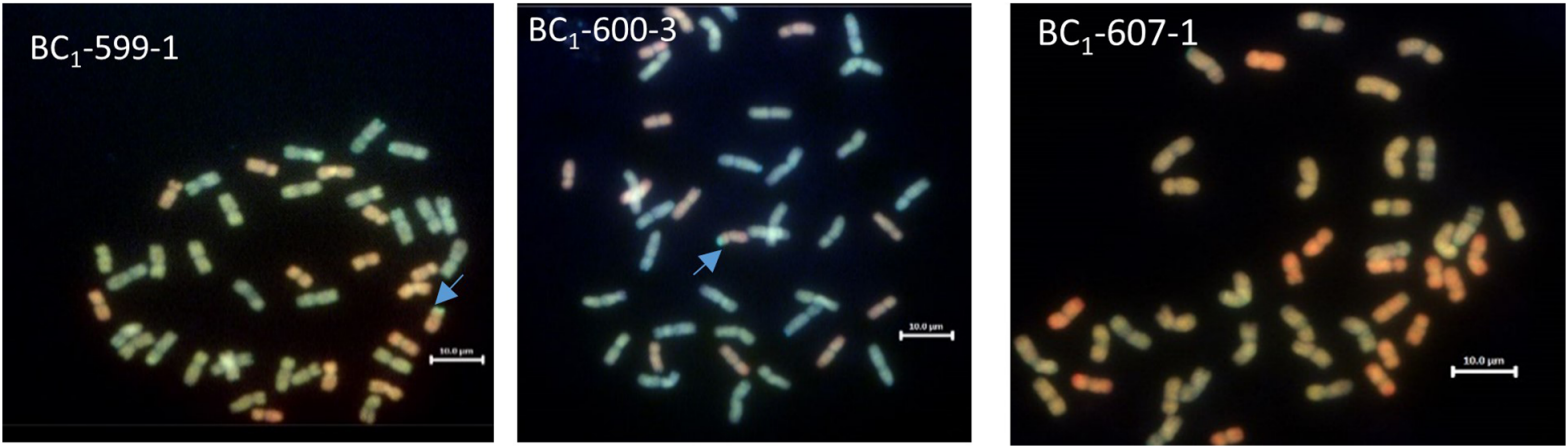
GISH images of BC_1_ root metaphase spreads showing the A, B, D and T genomes (A genome - green, B genome - blue, D genome – red, T genome - gold). The blue arrows indicate the site of *Am. muticum* (T) introgressions into Chr 4D of wheat. GISH image for line BC_1_-607-1 shows a plant where KASP showed a segment on wheat chromosome 7A, and GISH showed no segment present.

**Figure 4:**
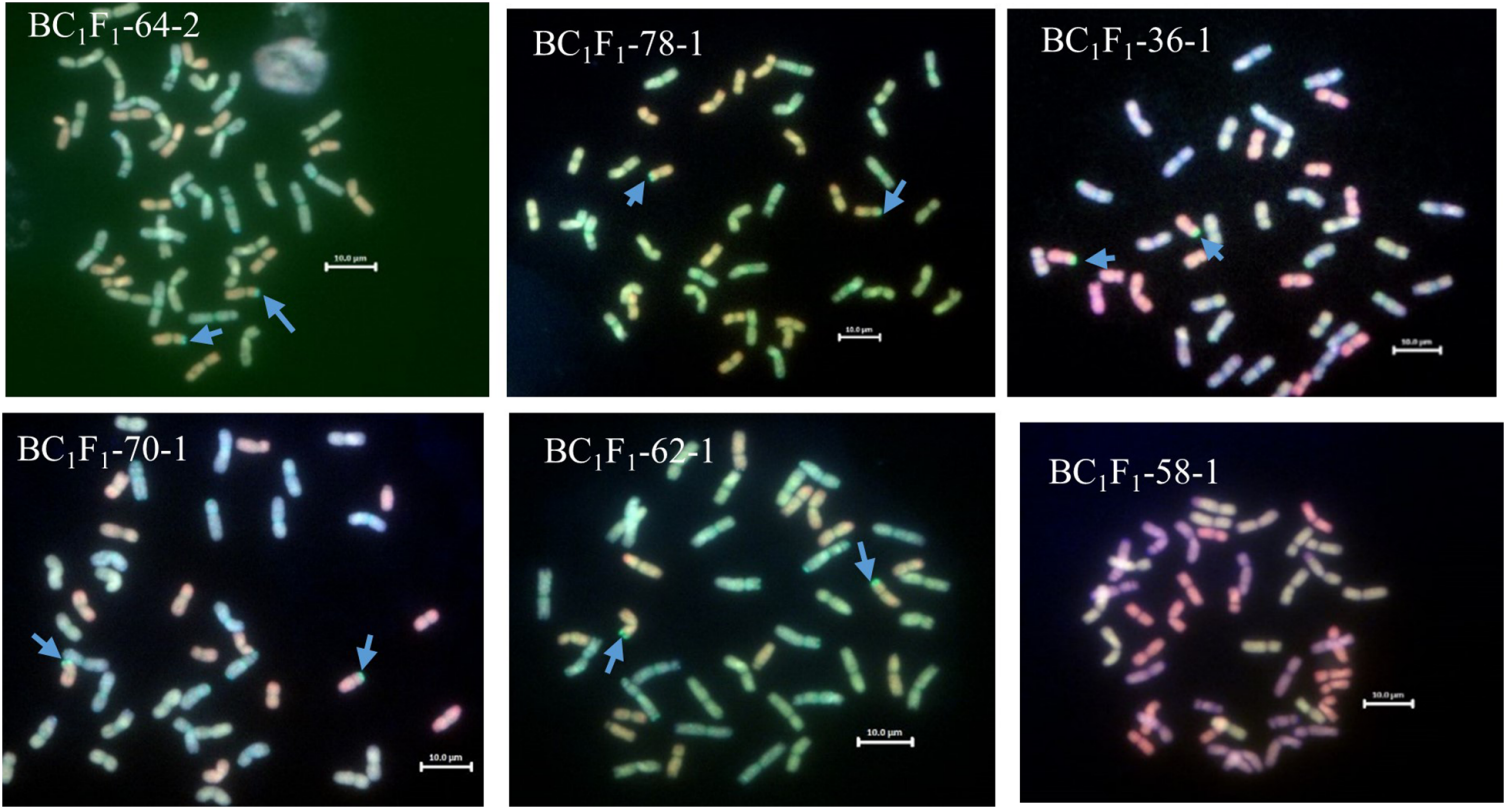
GISH images of BC_1_F_1_ root metaphase spreads showing the A, B, D and T genomes (A genome - green, B genome - blue, D genome – red, T genome - gold). The blue arrows indicate the site of *Am. muticum* (T) introgressions into Chr 4D of wheat. BC_1_F_1_-64-2 shows only a pair of 4T segments on wheat Chr 4D, GISH did not capture the other set of 7T segments revealed by KASP. BC_1_F_1_-78-1, BC_1_F_1_-36-1 and BC_1_F_1_-70-1 show euploid sets of chromosomes each with a pair of 4T segments on wheat Chr 4D. BC_1_F_1_-62-1 shows an anueploid (41 with a missing D chromosome) metaphase spread with a pair of 4T segments on Chr 4D. BC_1_F_1_-58 has a 7A segment undetectable by GISH; however, this shows an aneuploid set of chromosomes (43 with an extra B chromosome).

One hundred and eighty-two chromosome-specific KASP markers were tested on the parental line, three of the *Am. muticum* DH-348, three of the *Am. muticum* accession 2130012 and the three Malawian wheat varieties. Eight to ten markers were selected for each linkage group (1A-7A, 1B-7B and 1D-7D) based on their position and results from previous work (King et al., 2019). The markers were designated codes between WRC1001-WRC1329 (Grewal et al., 2020) and WRC1330-WRC169 (Grewal et al., 2022). Genotyping of 80 wheat/*Am. muticum* BC_1_ plants with group 4 (WRC1314, WRC1315, WRC1316 and WRC1784) and group 7 markers (WRC2020 and WRC2104), detected the presence of heterozygous *Am. muticum* segments on wheat Chr 4D and Chr 7A in 19 lines, the 4D segment in 15 lines, and the 7A segment in 13 lines. To validate the genotyping results, multi-colour GISH was performed on 40 of 47 BC_1_ plants heterozygous for the *Am. muticum* segments. GISH analysis of the 19 lines with the 4T and 7T segments validated the presence of a heterozygous segment on the distal end of the short arm of wheat Chr 4D but not the 7T segment because of the small size (**Figure 3**). Lines with only the 4T segment also showed heterozygous segments on wheat chromosome 4D, whilst lines with only the 7A segment were not verified by GISH. Subsequent genotyping of 85 BC_1_F_1_ plants obtained from selfing the heterozygous BC1 population detected 25 lines homozygous for the 4T segment on Chr 4D, 14 lines homozygous for the 7T segment on Chr 7A and 2 lines (BC_1_F_1_ 64-2 and BC_1_F_1_ 61-2) with both the segments on Chr 7A and Chr 4D homozygous. Further analysis showed that 26 lines remained heterozygous for the segment on Chr 4D while the rest of the lines had lost the segments (**Table 1**). To validate these results mcGISH was performed on 31 of the 41 BC_1_F_1_ plants homozygous for the *Am. muticum* segments. The presence of a pair of 4T segments in the lines observed with only a 4T/4D recombination was also validated in 23 of the 25 introgression lines, and no segment was detected in seven of the fourteen lines with the 7T/7D recombination. In the lines with both the 4T and 7T segments, GISH validated the presence of a pair of the 4T in line BC_1_F_1_ 64-2 whilst line BC_1_F_1_ 61-2 could not be validated, because the roots obtained did not give good metaphase spreads. GISH also showed that 25 of the 31 BC_1_F_1_ lines analysed had maintained the euploid chromosome condition, while five lines had a missing D chromosome, likely inherited from the monosomic deletion observed in the sequence of the parental line DH-348. Line BC_1_F_1_ 58-1 showed the entire chromosome set, plus an extra B chromosome (**Figure 4**).

**Table 1:** A list of Malawian wheat/*Am. muticum* DH-348 BC_1_ and BC_1_F_1_ lines showing number of *Am. muticum* segments detected by chromosome specific KASP markers and verified by GISH, location of the segments on the wheat chromosomes and number of chromosomes revealed by GISH analysis.

Cross combination	BC_1_ code	BC_1_F_1_ code	No. of segments KASP	No of segments GISH	Location on wheat chromosome	No. of chromosomes	Missing/extra
DHF1 348 x Nduna	BC_1_ 605-2	BC_1_F_1_ 64-2	2	1	4D,7A	42	0
	BC_1_ 606-1	BC_1_F_1_ 62-1	1	1	4D	41	D
	BC_1_ 603-3	BC_1_F_1_ 67-4	1	0	7A	42	0
	BC_1_ 606-3	BC_1_F_1_ 63-2	1	1	4D	42	0
	BC_1_ 603-3	BC_1_F_1_ 67-2	1	1	4D	42	0
DHF1 348 x Kadzibonga	BC_1_ 597-2	BC_1_F_1_ 78-1	1	1	4D	42	0
	BC_1_ 599-1	BC_1_F_1_ 72-2	1	1	4D	42	0
	BC_1_ 600-3	BC_1_F_1_ 70-1	1	1	4D	42	0
	BC_1_ 600-1	BC_1_F_1_ 71-1	1	0	7A	42	0
	BC_1_ 600-1	BC_1_F_1_ 71-3	1	0	7A	–	–
	BC_1_ 598-3	BC_1_F_1_ 75-1	1	0	7A	–	–
	BC_1_ 599-4	BC_1_F_1_ 73-2	1	1	4D	–	–
	BC_1_ 600-4	BC_1_F_1_ 123-3	1	0	7A	-	–
Nduna x DHF1 348	BC_1_ 607-4	BC_1_F_1_ 60-1	1	1	4D	42	0
	BC_1_ 607-4	BC_1_F_1_ 60-2	1	1	4D	42	0
	BC_1_ 608-3	BC_1_F_1_ 59-2	1	1	4D	42	0
	BC_1_ 608-2	BC_1_F_1_ 58-1	1	0	7A	43	B
	BC_1_ 610-1	BC_1_F_1_ 54-1	1	0	7A	–	–
	BC_1_ 607-3	BC_1_F_1_ 61-2	2	1	4D,7A	–	–
	BC_1_ 609-3	BC_1_F_1_ 56-1	1	1	4D	–	–
	BC_1_ 609-3	BC_1_F_1_ 56-2	1	1	4D	41	D
	BC_1_ 605-3	BC_1_F_1_ 113-2	1	1	4D	42	0
	BC_1_ 607-1	BC_1_F_1_ 116-2	1	0	7A	42	0
	BC_1_ 606-1	BC_1_F_1_ 62-1	1	1	4D	42	0
	BC_1_ 606-1	BC_1_F_1_ 62-3	1	0	7A	42	0
Kadzibonga x DHF1 348	BC_1_ 612-3	BC_1_F_1_ 50-1	1	1	4D	42	0
	BC_1_ 611-1	BC_1_F_1_ 51-1	1	1	4D	42	0
	BC_1_ 612-2	BC_1_F_1_ 49-1	1	1	4D	42	0
	BC_1_ 611-4	BC_1_F_1_ 53-1	1	0	7A	42	0
	BC_1_ 612-3	BC_1_F_1_ 50-2	1	1	4D	42	0
Kenya Nyati x DHF1 348	BC_1_ 618-1	BC_1_F_1_ 38-1	1	1	4D	42	0
	BC_1_ 615-3	BC_1_F_1_ 42-2	1	1	4D	41	D
	BC_1_ 616-2	BC_1_F_1_ 35-1	1	1	4D	42	0
	BC_1_ 616-2	BC_1_F_1_ 35-2	1	1	4D	41	D
	BC_1_ 616-3	BC_1_F_1_ 36-1	1	1	4D	42	0
	BC_1_ 617-1	BC_1_F_1_ 37-1	1	1	4D	41	D
	BC_1_ 618-3	BC_1_F_1_ 39-1	1	0	7A	42	0
	BC_1_ 615-4	BC_1_F_1_ 43-2	1	0	7A	42	0
	BC_1_ 615-4	BC_1_F_1_ 43-3	1	1	4D	42	0
	BC_1_ 615-6	BC_1_F_1_ 121-3	1	0	7A	–	–
	BC_1_ 615-1	BC_1_F_1_ 120-3	1	0	7A	–	–

Lines with a – on chromosome numbers were not verified with GISH.

### 3.4 Molecular and cytogenetic characterisation of Malawian wheat/*T. urartu* BC_1_ and BC_1_F_1_ plants with chromosome specific KASP markers

**Figure 5:**
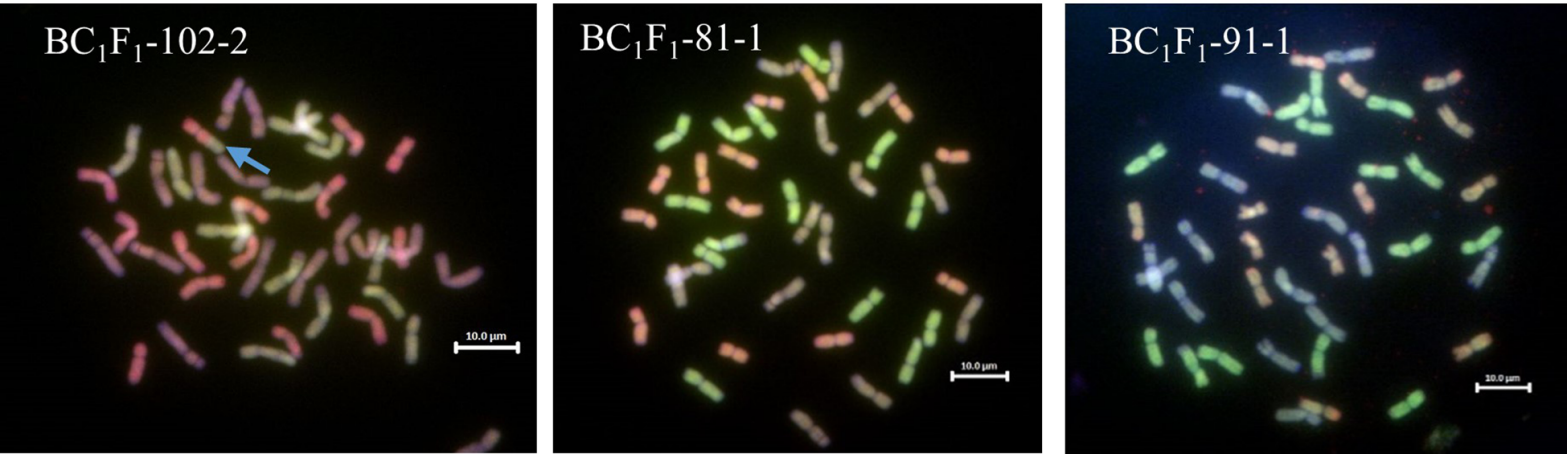
GISH images of metaphase spreads from the BC_1_F_1_ roots of Malawian wheat/*T. urartu* introgression lines showing the A, B and D genomes (A genome - green, B genome - blue, D genome – red). The blue arrow on BC_1_F_1_-102-2 shows the 5A-5D translocation.

Malawian wheat/*T. urartu* DH-254, three of the *T. urartu* accessions, three of the *T. urartu* DH-254 and the three Malawian varieties were genotyped using 144 chromosome-specific KASP markers polymorphic between wheat and *T. urartu.* Markers were selected based on their availability and results from the previous work (Grewal et al., 2018a). Genotyping of 35 BC_1_ plants with the group 5 markers within the region of the 76.40 Mbps segment (WRC605 and WRC608) gave heterozygous calls for the *T. urartu* segment on wheat Chr 5A in 31 lines and a homozygous wheat call on the remaining lines. The marker detecting the 5A^u^ smaller segment (28.7 Mbps) was unable to detect the DH-254 controls, and thus none of the BC_1_ plants could be scored for the small segment. Subsequent characterisation of 81 BC_1_F_1_ plants for the larger 5A^u^ segment detected 14 homozygous lines, 50 heterozygous lines and 21 lines with no segment (**Table 2**). Among the 14 homozygous lines, only 11 grew to maturity, produced seed, and could be carried forward to the next generation. GISH analysis of selected BC_1_ and BC_1_F_1_ did not validate the presence of the 5A^u^ segment recombined with the 5A chromosome of wheat. However, GISH detected the presence of the 5A-5D translocation initially observed in both the sequence visualisation and GISH image of the parental line (*T. urartu* DH-254). GISH analysis also revealed the number of chromosomes of all the introgression lines (**Figure 5**).

**Table 2:** A list of Malawian wheat/*T. urartu* DH 254 BC_1_ and BC_1_F_1_ lines showing number of *T. urartu* segments detected by KASP markers, their location on the wheat genome and number of chromosomes revealed by GISH analysis.

Cross combination	BC_1_ code	BC_1_F_1_ code	No of A^U^ segments by KASP	No of A^U^ segments by GISH	Location on wheat chromosome	No. of chromosomes
DHF1 254 x Kenya nyati	BC_1_ 642-5	BC_1_F_1_ 91-3	1		5A	–
	BC_1_ 642-2	BC_1_F_1_ 89-2	1	0	5A	42
	BC_1_ 640-4	BC_1_F_1_ 82-1	1	0	5A	13A,14B,13D+1A/D
	BC_1_ 640-5	BC_1_F_1_ 83-1	1	0	5A	42
	BC_1_ 642-5	BC_1_F_1_ 91-1	1	0	5A	42
	BC_1_ 640-2	BC_1_F_1_ 81-1	1	0	5A	41
	BC_1_ 640-2	BC_1_F_1_ 81-2	1	0	5A	41
	BC_1_ 644-4	BC_1_F_1_ 97-2	1	0	5A	42
DHF1 254 x Nduna	BC_1_ 647-5	BC_1_F_1_ 105-1	1		5A	–
	BC_1_ 647-1	BC_1_F_1_ 102-2	1	0	5A	14A,14B, 13D+1A/D
	BC_1_ 647-5	BC_1_F_1_ 105-3	1		5A	–
Kenya Nyati x DHF1 254	BC_1_ 649-4	BC_1_F_1_ 108-1	1		5A	–
	BC_1_ 649-1	BC_1_F_1_ 87-2	1		5A	–
	BC_1_ 648-2	BC_1_F_1_ 107-4	1		5A	–

Lines with a – on chromosome numbers were not verified with GISH.

### 3.5 Soil analysis

**Table 3** describes the soil physio-chemical properties of the soils at the experimental site. The soils were classified as clay loam, with an average soil pH of 6.7. DTPA-Zn and Fe were 0.3 and 7.7 mg kg^-1^ respectively. Soil analysis also showed that the soil samples had an average of 0.2% total nitrogen, 20.6 mg kg^-1^ available P and 67.4 mg kg^-1^ K.

**Table 3:** Physio-chemical properties of soil samples collected from the three replicates of the experimental site.

Parameter	Block 1	Block 2	Block 3	Average
Soil pH	6.6	6.7	6.7	6.7
Organic matter (%)	2.0	2.9	2.3	2.4
DTPA-Zn (mg/kg)	0.2	0.4	0.3	0.3
DTPA-Fe (mg/kg)	7.2	8.3	7.7	7.7
Total N (%)	0.3	0.2	0.2	0.2
Available P (mg/kg)	20.3	20.6	21	20.6
K (mg/kg)	62.1	56.9	83.2	67.4
Silt (%)	16	14	16	15.3
Clay (%)	44	44	44	44.0
Sand (%)	42	40	40	40.7
Textural class	Clay loam	Clay loam	Clay loam	Clay loam

### 3.6 Mineral analysis

#### 3.6.1 Grain zinc concentration

Analysis of grain samples showed a significant variation in grain Zn concentrations (P<0.0001) among the 55 genotypes (**Table 4**). Grain Zn concentrations varied from 35.5-108.6 mg kg^-1^ with an overall mean of 57.9 mg kg^-1^. DH-348 had the highest grain Zn concentration of all the genotypes analysed with 108.6 mg kg^-1^, while BC_1_F_3_-30 had the highest grain Zn concentration of the BC_1_F_3_ introgression lines with 84.9 mg kg^-1^. Overall, 13% of the BC_1_F_3_ lines had Zn concentrations between 70-85 mg kg^-1^, 25% between 60-68 mg kg^-1^ and 43% between 50-59 mg kg^-1^. The three Malawian checks, *Kenya nyati*, *Kadzibonga* and *Nduna* had grain zinc concentrations of 42.0, 35.8 and 35.3 mg kg^-1^ respectively, and these were the lowest among all the genotypes, except for BC_1_F_3_-44, which had a concentration of 38.6 mg kg^-1^. Mineral analysis also showed a significant variation among the UK checks, with Pavon 76 having the highest concentration. Mineral analysis for *T. urartu* DH-254 was not conducted because the plants did not produce any seed.

**Table 4:** Variation in grain zinc and iron concentration and associated grain yield, yield components and phenotypic and phenological traits of 37 Malawian wheat/DH-348 and 11 Malawian wheat/DH-254 BC_1_F_3_ introgression lines grown in 2022 winter season.

Genotypes	Grain Zn (mg kg^-1^)	Grain Fe (mg kg^-1^)	Grain yield (kg/ha)	Thousand kernel weight (g)	Number of tillers	Plant Height (cm)	Days to heading	Days to anthesis	Days to maturity	Spike type
DH 348	108.6	95.8	729	35	7	54	105	109	135	Awnless
BC_1_F_3_-30	84.9	87.3	724	25	8	60	119	123	149	Awned
BC_1_F_3_-10	76.9	55	2889	50	5	55	67	71	97	Awned
BC_1_F_3_-39	76.6	59.2	2778	50	6	62	72	76	102	Awnless
BC_1_F_3_-13	75.9	76.7	1630	43	8	54	71	75	101	Awnless
BC_1_F_3_-11	73.2	70.7	1091	40	7	80	92	96	122	Awnless
BC_1_F_3_-27	73.1	66	2407	50	5	54	65	68	95	Awnless
BC_1_F_3_-28	73.1	66	1222	42	8	85	110	114	140	Awnless
BC_1_F_3_-34	67.1	48.1	4630	50	6	59	90	94	120	Awned
BC_1_F_3_-36	66.7	52	4148	50	7	62	67	71	97	Awned
BC_1_F_3_-38	65.7	49.9	3148	50	7	51	74	78	104	Awned
BC_1_F_3_-29	64.2	64	1014	40	7	71	98	102	129	Awnless
Pavon	63.9	61.4	2148	50	6	66	73	77	103	Awned
BC_1_F_3_-20	63.8	60.3	2370	50	4	45	66	70	96	Awned
BC_1_F_3_-32	63.8	48.9	2333	50	6	62	67	71	97	Awnless
BC_1_F_3_-42	63.3	48.9	1378	40	8	89	114	117	144	Awned
BC_1_F_3_-47	61	52.6	2630	42	8	74	119	123	149	Awned
BC_1_F_3_-40	60.8	47.7	3741	50	7	58	73	76	103	Awned
BC_1_F_3_-31	60.6	51.5	3037	50	7	48	78	72	108	Awned
BC_1_F_3_-37	60.4	51.5	4186	50	7	53	69	73	99	Awned
BC_1_F_3_-15	60.3	55.8	3037	50	6	51	68	73	98	Awned
BC_1_F_3_-18	59.9	51.2	3111	50	3	47	65	68	95	Awned
BC_1_F_3_-35	59.9	52.1	2326	49	7	64	72	76	102	Awned
BC_1_F_3_-16	59.7	53.4	2259	49	4	53	67	72	97	Awnless
BC_1_F_3_-46	58.9	45.1	4556	50	6	68	72	76	103	Awned
BC_1_F_3_-53	58.2	53.5	1815	43	9	73	119	123	149	Awned
BC_1_F_3_-57	57.7	56.1	2815	50	5	67	68	72	98	Awned
BC_1_F_3_-41	57.2	61.2	1804	51	7	70	73	77	103	Awned
BC_1_F_3_-45	57	56.6	1259	49	8	80	119	122	149	Awned
BC_1_F_3_-49	56.8	45.6	4741	55	8	61	68	71	98	Awned
BC_1_F_3_-19	55.8	54.8	3481	50	5	46	81	84	111	Awned
BC_1_F_3_-9	55.7	51.4	3333	52	5	58	77	81	107	Awned
BC_1_F_3_-2	55.4	59.3	1148	55	5	77	72	76	102	Awnless
BC_1_F_3_-33	53.5	56.3	2844	50	9	61	70	74	100	Awned
BC_1_F_3_-6	52.5	38.8	3259	50	6	62	86	90	116	Awnless
BC_1_F_3_-3	52.4	52.7	2333	50	6	74	81	85	113	Awnless
BC_1_F_3_-60	52	56	1000	44	7	73	94	98	132	Awned
BC_1_F_3_-26	51.9	66.2	2617	50	10	43	123	128	153	Awned
BC_1_F_3_-1	51.9	57.2	2148	48	5	52	72	76	102	Awned
BC_1_F_3_-48	50.3	45.7	1688	50	5	82	67	72	97	Awned
BC_1_F_3_-52	50	45.5	2741	50	3	49	65	68	95	Awned
BC_1_F_3_-7	49.5	54.7	2778	50	6	68	89	93	119	Awned
BC_1_F_3_-51	49.2	38	3074	50	6	58	65	69	95	Awned
BC_1_F_3_-23	48.4	46.2	1815	42	3	53	70	77	103	Awned
Chinese spring	48.2	60.4	1222	48	13	100	87	91	117	Awnless
BC_1_F_3_-21	47.4	53.4	2074	43	8	51	70	73	100	Awned
BC_1_F_3_-50	45	49	1556	50	9	72	83	97	113	Awned
BC_1_F_3_-17	44.4	517	3667	50	4	50	65	69	95	Awnless
BC_1_F_3_-5	44.4	51.7	2962	50	6	63	89	93	119	Awnless
BC_1_F_3_-54	43.2	51.7	815	45	12	72	75	79	104	Awned
Paragon	43	46.6	890	18	8	64	106	110	136	Awnless
Kenya Nyati	42	43.1	3037	50	6	56	69	72	99	Awned
BC_1_F_3_-44	38.6	45.9	2852	51	7	67	72	66	103	Awned
Kadzibonga	35.8	53.2	3111	49	3	57	81	85	111	Awnless
Nduna	35.3	41.3	3185	50	6	55	68	72	98	Awned
Grand mean	57.9	54.4	2448	47	6	63	81	85	111	
P- Value	<0.0001	<0.0001	<0.0001	<0.0001	<0.0001	<0.0001	<0.0001	<0.0001	<0.0001	
LSD (5%)	16.4	9.8	1455	8.5	3.2	14.1	19.7	20.3	19.7	
CV%	17.4	11.1	20.2	11.1	31	13.9	15.0	14.7	10.9	

Degrees of freedom (df) for replicates = 2, df for genotypes = 54.

The introgression lines have been ordered according to grain Zn (highest to lowest).

#### 3.6.2 Grain iron concentration

Significant variation (P<0.0001) was observed in the grain Fe concentrations of the 55 genotypes (**Table 4**). The Fe concentrations varied from 38.3-95.8 mg kg ^-1^ with an overall mean of 54.2 mg kg^-1^. DH-348 showed the highest grain Fe concentration (95.8 mg kg ^-1^) followed by BC_1_F_3_-30 (87.4 mg kg ^-1^), BC_1_F_3_-13 (76.6 mg kg^-1^) and BC_1_F_3_-11 (70.7 mg kg^-1^) respectively. Overall, 6% of the BC_1_F_3_ introgression lines had Fe concentrations between 70-87 mg kg ^-1^, 10% between 60-66 mg kg^-1^, 54% between 50-59 mg kg ^-1^, and the remaining had Fe concentrations above 40 mg kg^-1^, with the exceptions of BC_1_F_3_-6 and BC_1_F_3_-51, which had 38.8 and 38.3 mg kg^-1^. The Fe concentrations of the Malawian checks, *Kadzibonga*, *Kenya nyati* and *Nduna* were 53.2, 42.0 and 35.3 mg kg^-1^ while Pavon 76, Chinese spring and Paragon had Fe concentrations of 61.4, 60.4 and 46.6 mg kg^-1^ respectively.

### 3.7 Grain yield and associated traits

Grain yields varied significantly (P< 0.0001), ranging from 724-5741 kg ha^-1^, with an overall mean of 2448 kg ha^-1^ (**Table 4**). BC_1_F_3_-49, BC_1_F_3_-34 BC_1_F_3_-46, BC_1_F_3_-37, BC_1_F_3_-36, BC_1_F_3_-40, BC_1_F_3_-17, BC_1_F_3_-19, BC_1_F_3_-9 and BC_1_F_3_-6 yielded 4741, 4630, 4556, 4186, 4148, 3741, 3667, 3481, 3333 and 3259 kg ha^-1^ respectively. The yields of these 10 lines were higher than the highest yielding Malawian check *Nduna*, which had a yield of 3185 kg ha^-1^. Although *Nduna* showed the highest yield among the Malawian checks, *Kadzibonga* and *Kenya nyati*, and introgression lines BC_1_F_3_-38, BC_1_F_3_-18, BC_1_F_3_-51, BC_1_F_3_-15 and BC_1_F_3_-31 had statistically similar yields. Paragon had the lowest grain yield (890 kg ha^-1^) out of the three UK checks, while Pavon 76 and Chinese Spring had 1222 and 2148 kg ha^-1^ respectively. Grain yields for BC_1_F_3_-30 and DH-348 were 729 and 724 kg ha^-1^, and these were the lowest yields among all the genotypes. The thousand kernel weight varied (P<0.0001) from 18-55 g, with an overall mean of 47 g. BC_1_F_3_-49 and BC_1_F_3_-2 had the highest kernel weight (55 g each), followed by BC_1_F_3_-9 and BC_1_F_3_-26, both with 52 g. TKW for the majority of the BC_1_F_3_ introgression lines was 50 g and this was statistically comparable to all three Malawian checks, Pavon 76, and Chinese Spring. DH-348, BC_1_F_3_-30 and Paragon had the lowest TKW among all the genotypes. Significant variation (P<0.0001) was also observed in the days to flowering, days to heading and days to maturity (**Table 4**). Days to heading varied from 65-123, days to flowering 69-128 while days to maturity varied from 95-153. BC_1_F_3_- 26 BC_1_F_3_- 55 BC_1_F_3_-30, BC_1_F_3_-53, BC_1_F_3_-47 and BC_1_F_3_-45 had the longest time to heading, flowering and maturing, while BC_1_F_3_-18, BC_1_F_3_-52, BC_1_F_3_- 17, BC_1_F_3_-27 and BC_1_F_3_-51 took the shortest time. The majority of the BC_1_F_3_ introgression lines took 65-80 days to heading, 69-85 days to flowering, and 95-108 days to maturing. The number of tillers were significantly variable (P< 0.0001), varying from 3-13 with an overall mean of six. Chinese Spring had the highest number of tillers (13), followed by BC_1_F_3_-54 with 12 tillers. Among the introgression lines, 22, 20, 18 and 16% had seven, six, eight and five tillers respectively. Plant height of the 55 genotypes was also highly significant (P<0.0001), ranging from 33-100 cm. Plant height for the majority of the BC_1_F_3_ lines varied from 50-70 cm, with a few lines between 71-89 cm. Chinese Spring grew to 100 cm, whilst heights of Paragon and Pavon 76 were 64 and 66 cm, respectively. *Nduna*, *Kenya nyati* and *Kadzibonga* had heights of 55, 56 and 57 cm, respectively. It was observed that amongst the BC_1_F_3_ introgression lines, 72% of the spikes had awns while 28% were awnless. Among the checks, Paragon, Chinese Spring, DH-348 and *Kadzibonga* had awnless spikes, while Pavon 76, *Kenya nyati* and *Nduna* showed awned spikes.

### 3.8 Correlation analysis

**Figure 6:**
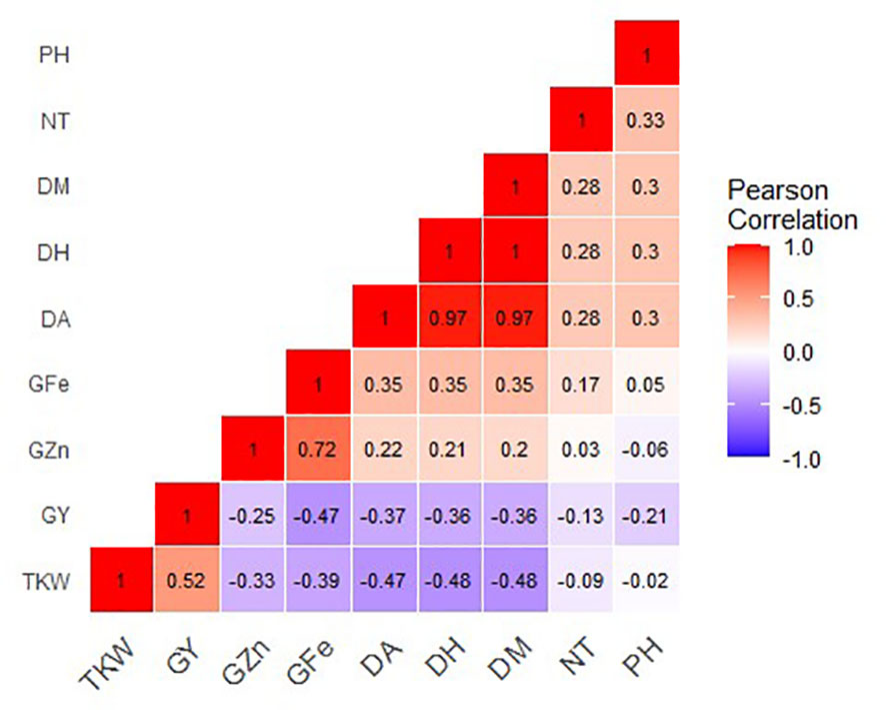
Correlation coefficients for grain mineral-elements and phenotypic and phenological data of 37 Malawian wheat/*Am. muticum* and 11 Malawian wheat/*T. urartu* BC_1_F_3_ introgression lines grown in 2022 winter season. G Zn, grain zinc; G Fe, grain iron; NT, number of tillers; DH, days to heading; DF, days to flowering; PH, plant height; TKW, thousand kernel weight; GY, grain yield.

Grain Zn concentration positively and significantly correlated with grain Fe (r = 0.72, P =<0.0001. However, grain Zn showed a weak negative correlation with both TKW (r = -0.33, P<0.0001) and grain yield (r = -0.25, P = 0.009). Grain Fe showed a weak positive correlation with days to heading (r = 0.35, P< 0.0001), days to anthesis (r = 0.35, P<0.0001) and days to maturity (r = 0.35, P< 0.0001). Correlation analysis also showed that grain Fe negatively and significantly correlated with both TKW (r = -0.39, P<0.0001), and grain yield (r = 0.47, P<0.0001). TKW positively correlated with Grain yield (r = 0.530, P<0.0001), and there were strong positive correlations between days to heading, flowering and maturity (**Figure 6**).

## 4 Discussion

The use of chromosome introgressions from distantly related or unrelated species that carry genetic variation for high mineral concentration of essential elements is one of the approaches that can be utilised to increase micronutrient concentration in crops (Velu et al., 2019). Recently, high Zn wheat varieties developed from crossing the wheat progenitor *Ae. tauschii* with *T. durum*/wild tetraploid *T. dicoccum* via synthetic wheat, were released in Pakistan and India (Singh et al., 2017; Velu et al., 2019). Screening of rye translocation lines in a wheat backgrounds also showed significantly higher Zn and Fe concentration above their recurrent parents (Velu et al., 2019). At the Nottingham WRC, a number of *Am*. *muticum* and *T. urartu* introgression lines developed for trait analysis were shown to have potential for increased grain Zn and Fe above their recurrent parents, and T. *urartu*, DH-254 and *Am. muticum*, DH-348 showed the highest grain Zn and Fe concentrations (Guwela, 2023; Guwela et al., 2024). The two lines were therefore selected for the current study based on their high Zn and Fe concentrations.

To transfer the *Am. muticum* (TT) and *T. urartu (*A^u^A^u^ *)* introgressions, which potentially increased mineral nutrients, from the DH lines into Malawian wheat varieties, hexaploid wheat/A*m. muticum* DH-348 and hexaploid wheat/*T. urartu* DH-254 were crossed with the three Malawian wheat varieties (*Kadzibonga*, *Nduna* and *Kennya nyati*). A combination of whole genome sequencing, KASP analysis and genomic *in situ* hybridisation (GISH) revealed a 4T and a 7T segment of *Am. muticum* on wheat chromosome 4D and 7A of *Am. muticum* DH-348. Whole genome sequencing and KASP analysis also revealed the presence of two 5A^u^ segments on wheat chromosome 5A of *T. urartu* DH-254. A crossing program for *Am. muticum* DH-348 and *T. urartu* DH-254 with *Kadzibonga*, *Nduna* and *Kenya nyati* resulted in the generation of forty-one Malawian wheat/*Am. muticum* BC_1_F_3_ introgression lines with both the 4T and 7T segments, 4T segments only, and 7T segments only. Eleven Malawian wheat/*T. urartu* BC_1_F_3_ introgression lines with the 5A^u^ segment were also generated. The availability of high-throughput genotyping technologies has enabled the process of tracking wild chromosome segments in a wheat genetic background. Through a combination of whole genome sequencing, KASP genotyping with chromosome specific markers and GISH, a clear picture of the genetic make-up of the donor parents was revealed. This made it easier to track the chromosome segments though the breeding pedigree.

The BC_1_F_3_ introgression lines carrying *Am. muticum* and *T. urartu* chromosome segments in the three Malawian wheat genetic backgrounds were phenotyped for grain Zn and Fe concentrations, and related agronomic traits under field conditions in Malawi. Soil samples collected at the trial site showed that the soils could be classified as Zn-deficient (Noulas et al., 2018; De Groote et al., 2021). Grain Zn concentration varied widely among the introgression lines, with 98% (47) of the lines showing a grain Zn concentration above Chinese Spring, Paragon (the wheats in the background of DH-348), and the three recurrent parents/Malawian checks (*Kenya nyati*, *Kadzibonga* and *Nduna*), and 80% (38) of these improved in grain Zn concentration up to 50% above *Kadzibonga* and *Nduna*. Although 10% of the BC_1_F_3_ introgression lines had grain Zn between 70-80 mg kg ^-1^, all of them had lower yields than the potential yield of the three Malawian checks (~3000 kg ha^-1^). However, one line (BC_1_F_3_-10) had a grain yield only slightly lower (2889 kg ha^-1^) than the Malawian checks, with a good combination of increased grain Zn and Fe concentrations. The number of crosses could have affected total grain yield of the introgression lines. Due to limited time, the two DH lines had only been crossed twice to the Malawian genotypes. Therefore, a quarter of the background of the introgression lines was still Chinese spring/Paragon, which are not adapted to Malawian conditions. Crossing the most promising lines four more times with the Malawian wheat varieties to remove all Chinese Spring/Paragon from the background will likely improve their yields/agronomic performance. Previous studies have also shown trade-offs between grain yield and grain Zn concentration (Liu et al., 2014; Gashu et al., 2021; Velu et al., 2022; Hasheminasab et al., 2023). Xia et al. (2023) suggested that improving agronomic management, including appropriate N fertilization and rotation can achieve high yield while ameliorating the dilution of grain Zn density in wheat. A global meta-analysis of the effects of nitrogen fertilization effects on grain Zn and Fe of major cereal crops also revealed that trade-offs between grain Zn and Fe and grain yield were higher at lower N application compared to higher N application (Zhao et al., 2022). 23% of the introgression lines (BC_1_F_3_-34, BC_1_F_3_-36, BC_1_F_3_-38, BC_1_F_3_-40, BC_1_F_3_-31, BC_1_F_3_-37, BC_1_F_3_-15, BC_1_F_3_-46, BC_1_F_3_-19, BC_1_F_3_-9 and BC_1_F_3_-6) showed a good combination of grain Zn concentration and grain yield. Grain yield of these lines was similar or exceeded most of the local checks, ranging from 3037 to 4630 kg ha^-1^, with Zn concentration ranging from 53-67 mg kg^-1^, which represents a 16-30 mg kg^-1^ improvement in grain Zn from *Nduna* and *Kadzibonga* and 11-25 mg kg^-1^ from *Kenya nyati*, Paragon and Chinese Spring. Interestingly, 10 of the 11 lines were awned, with a maturity period between 97-120 days, making them more suited to the SSA environments. Ten of the 11 lines carry either the 4T or 7T segments from *Am. muticum*, and only one carries the 5A^u^ segment from *T. urartu*. Although most of the lines with the *T. urartu* had increased grain Zn concentrations, most of them were long duration with yields much lower than the Malawian checks. This could be an effect of the size of the 5A^u^ segment, carrying along genes that negatively affect the performance of the introgression lines. During the period of crossing, the *T. urartu* donor parent (DH-254) was shown to have longer days to heading and flowering, which affected the number of crosses made, as the heading and anthesis did not coincide with that of the early maturing recurrent parents. Among the 23% (11) high Zn, high yield introgression lines, 64% (7) lines also had an 8-12 mg kg ^-1^ higher Fe concentration than the recurrent parents *Nduna* and *Kenya nyati*, although they did not hit the HarvestPlus target for Fe biofortification in wheat (60 mg kg ^-1^). Of the 48 BC_1_F_3_ introgression lines, only nine lines reached ~60 mg kg ^-1^. However, the yields of the lines were much lower (< 2000 kg ha^-1^) than the yields of the Malawian checks. Although previous studies in wheat and other cereals have shown very low positive or a negative correlation between grain Zn and Fe (Morgounov et al., 2007; Joshi et al., 2010; Kanatti et al., 2014), this study showed a significant positive correlation between the two variables implying that the two can be improved simultaneously. Similar findings were previously reported (Velu et al., 2011; Crespo-Herrera et al., 2016; Velu et al., 2019; Thapa et al., 2022; Velu et al., 2022). The significant negative correlations between grain Zn and TKW/yield and Fe and TKW/yield implies that an increase in Zn and Fe concentration is associated with decreased TKW and yields. Similar results were reported previously (Velu et al., 2011; Liu et al., 2014; Velu et al., 2019; Thapa et al., 2022; Velu et al., 2022). Liu et al. (2014) showed that for every 1000 kg ha^−1^ increase in grain yield, Fe concentration decreased by 2.1 mg kg^−1^ for spring wheat, and Zn concentration decreased by 0.9 mg kg^−1^ due to dilution effects. The positive weak correlation between grain Fe and Zn concentration with crop phenological traits (days to heading, anthesis and maturity) tends to be weak, suggesting near-independence of these traits.

## 5 Conclusion

The results in this study show the possible significant impact of the 4T and 7T introgressions from *Am. muticum* and the 5A^u^ introgression from *T. urartu* on the genetic biofortification of Malawian wheat varieties particularly with higher grain Zn and Fe concentration. Identifying candidate genes associated with the high accumulation of grain Zn and Fe will be useful for future work. Currently, sequencing of *Am*. *muticum* accessions and hexaploid wheat/*Am*. *muticum* introgressions lines are being undertaken at the University of Nottingham WRC. These will play a major role in gene identification. Further testing of introgression lines in replicated and multi-location trials will also be useful to measure stability, heritability, yields and other important agronomic traits. To date, early trait analyses of a small selection of lines carrying different *Am. muticum* and *T. urartu* introgressions have already revealed critical genetic variation for a range of traits. These include resistances to a range of diseases including all three wheat rusts in Am. muticum (Fellers et al., 2020), Septoria resistance and powdery mildew resistance, flowering morphology, and increased yield. These initial pilot experiments indicate the significant potential of the genetic variation that is carried by these wild relatives for future wheat improvement.

## Data availability statement

The original contributions presented in the study are publicly available. This data can be found here:PRJEB71366 and PRJEB70905/European Nucleotide Archive (ENA).

## Author contributions

VG: Conceptualization, Data curation, Formal analysis, Investigation, Methodology, Software, Visualization, Writing – original draft. MM: Conceptualization, Project administration, Supervision, Visualization, Writing – review & editing. MBr: Conceptualization, Funding acquisition, Methodology, Resources, Supervision, Validation, Visualization, Writing – review & editing. MH: Conceptualization, Methodology, Supervision, Validation, Visualization, Writing – review & editing. JB: Conceptualization, Methodology, Supervision, Validation, Visualization, Writing – review & editing. SG: Writing – review & editing, Software, Methodology, Formal analysis, Data curation, Conceptualization, Supervision, Visualization. BC: Software, Methodology, Formal analysis, Data curation, Writing – review & editing, Investigation. AH: Writing – review & editing, Visualization, Supervision, Methodology, Data curation, Validation. CY: Writing – review & editing, Methodology, Supervision, Visualization. MBa: Writing – review & editing, Data curation, Investigation. LW: Writing – review & editing, Visualization, Supervision, Methodology, Formal analysis. JK: Conceptualization, Funding acquisition, Investigation, Methodology, Project administration, Resources, Supervision, Validation, Visualization, Writing – review & editing.
